# Anti-*Trypanosoma cruzi* Properties of Sesquiterpene Lactones Isolated from *Stevia* spp.: In Vitro and In Silico Studies

**DOI:** 10.3390/pharmaceutics15020647

**Published:** 2023-02-15

**Authors:** Jimena Borgo, Orlando G. Elso, Jessica Gomez, Mauro Coll, Cesar A. N. Catalán, Juan Mucci, Guzmán Alvarez, Lía M. Randall, Patricia Barrera, Emilio L. Malchiodi, Augusto E. Bivona, María Florencia Martini, Valeria P. Sülsen

**Affiliations:** 1 Instituto de Química y Metabolismo del Fármaco (IQUIMEFA), CONICET-Universidad de Buenos Aires, Junín 956, piso 2, Buenos Aires C1113AAD, Argentina; 2 Cátedra de Farmacognosia, Facultad de Farmacia y Bioquímica, Universidad de Buenos Aires, Junín 956, piso 2, Buenos Aires C1113AAD, Argentina; 3 Unidad de Microanálisis y Métodos Físicos Aplicados a Química Orgánica (UMYMFOR), Facultad de Ciencias Exactas y Naturales, CONICET-Universidad de Buenos Aires, Ciudad Universitaria, Pabellón 2, piso 3, Buenos Aires C1428EGA, Argentina; 4 Facultad de Ciencias Médicas, Instituto de Histología y Embriología “Dr. Mario H. Burgos” (IHEM), Universidad Nacional de Cuyo-CONICET, CC 56, Mendoza 5500, Argentina; 5 Instituto de Química Orgánica, Facultad de Bioquímica, Química y Farmacia, Universidad Nacional de Tucumán, Ayacucho 471, San Miguel de Tucumán, Tucumán T4000INI, Argentina; 6 Instituto de Investigaciones Biotecnológicas, Universidad Nacional de San Martín-CONICET, Buenos Aires 5500, Argentina; 7 Laboratorio de Moléculas Bioactivas, Departamento de Ciencias Biológicas, CENUR Litoral Norte, Universidad de la República, Paysandú 60000, Uruguay; 8 Cátedra de Inmunología, Facultad de Farmacia y Bioquímica, Universidad de Buenos Aires, Junín 956, piso 4, Buenos Aires C1113AAD, Argentina; 9 Instituto de Estudios de la Inmunidad Humoral (IDEHU), CONICET-Universidad de Buenos Aires, Junín 956, piso 4, Buenos Aires C1113AAD, Argentina; 10 Cátedra de Química Medicinal, Facultad de Farmacia y Bioquímica, Universidad de Buenos Aires, Junín 956, Planta Principal, Buenos Aires C1113AAD, Argentina

**Keywords:** sesquiterpene lactones, *Stevia satureiifolia* var *satureiifolia*, *Stevia alpina*, santhemoidin C, 2-oxo-8-deoxyligustrin, trypanocidal activity, molecular targets, prolyl oligopeptidase

## Abstract

*Stevia* species (Asteraceae) have been a rich source of terpenoid compounds, mainly sesquiterpene lactones, several of which show antiprotozoal activity. In the search for new trypanocidal compounds, *S. satureiifolia* var. *satureiifolia* and *S. alpina* were studied. Two sesquiterpene lactones, santhemoidin C and 2-oxo-8-deoxyligustrin, respectively, were isolated. These compounds were assessed in vitro against *Trypanosoma cruzi* stages, showing IC_50_ values of 11.80 and 4.98 on epimastigotes, 56.08 and 26.19 on trypomastigotes and 4.88 and 20.20 µM on amastigotes, respectively. Cytotoxicity was evaluated on Vero cells by the MTT assay. The effect of the compounds on trypanothyone reductase (TcTR), *Trans*-sialidase (TcTS) and the prolyl oligopeptidase of 80 kDa (Tc80) as potential molecular targets of *T. cruzi* was investigated. Santhemoidin C inhibited oligopeptidase activity when tested against recombinant Tc80 using a fluorometric assay, reaching an IC_50_ of 34.9 µM. Molecular docking was performed to study the interaction between santhemoidin C and the Tc80 protein, reaching high docking energy levels. Plasma membrane shedding and cytoplasmic vacuoles, resembling autophagosomes, were detected by transmission microscopy in parasites treated with santhemoidin C. Based on these results, santhemoidin C represents a promising candidate for further studies in the search for new molecules for the development of trypanocidal drugs.

## 1. Introduction

The *Stevia* genus (Asteraceae) comprises more than 230 endemic plant species, which are distributed from the southern United States to the Andean region of South America, to northern Chile, and to northern Patagonia in Argentina [1]. Twenty-nine medicinal species used in traditional medicine as well as other popular uses have been reported since the 18th century [2]. These plants have been associated with pharmacological properties such as antidiarrheal, anti-inflammatory, antimalarial, febrifuge, diuretic, diaphoretic and even aid the treatment of heart diseases, stomachaches, and skin conditions [3].

Sesquiterpene lactones (STLs) and diterpenoids are the two most characteristic groups of phytochemicals isolated from *Stevia* species [4]. Sesquiterpene lactones are 15-carbon terpenoid compounds that contain a lactone ring conjugated to an exomethylene group (α-methylene-γ-lactone). STLs have been thoroughly studied for their wide range of biological activities [5]. Their potential as antiprotozoal agents has been demonstrated in recent years by several authors [6,7,8,9].

Chagas disease, also known as American trypanosomiasis, is a parasitic disease caused by the protozoan *Trypanosoma cruzi*. It is considered one of the 20 neglected tropical diseases by the World Health Organization, since it mostly affects impoverished communities in tropical areas [10]. Approximately 6–7 million people worldwide carry the disease, which is endemic in 21 countries in Latin America and produces devastating health, social and economic consequences [11,12]. Available drugs for the treatment of this parasitosis are benznidazole and nifurtimox, which are effective in the acute phase of the infection, but they have limited efficacy for the treatment of the disease in the chronic stage [13,14] and are frequently associated with undesirable side effects; therefore, new treatments against Chagas are urgently needed [15].

Trypanosomatids maintain the cellular redox balance through the exclusive trypanothione metabolic system, which is vital for these parasites and is absent from the mammalian host, resulting in a metabolic pathway of interest in the development of effective and safe anti-*T. cruzi* drugs [16]. Trypanothione reductase (TcTR) is a key enzyme in the trypanothione metabolic system of *T. cruzi* and different authors have previously suggested it as a target of STLs [17,18].

*Trypanosoma cruzi* is incapable of synthesizing sialic acid (SA) de novo. Consequently, the expression of the *trans*-sialidase (TcTS) enzyme allows the cleavage of terminal SA residues present in glycoconjugates of host tissues. The SA obtained from this process is afterwards transferred onto mucins on the parasite surface, creating a protective and adhesive coat against the immune system. Additionally, TcTS shedding into the bloodstream induces alterations in the sialylation pattern of host cells, generating immune dysfunction and hematological alterations [19,20,21]. TcTS represents a potentially attractive drug target against *T. cruzi* since it is absent in mammalian hosts and because of its role in parasite survival.

During Chagas’ infection, parasite dissemination and invasion are related to its capacity to migrate through the extracellular matrix. The prolyl oligopeptidase of 80 kDa (Tc80) could facilitate invasion, given its ability to hydrolyze human type I and IV collagens. Parasitic and human prolyl oligopeptidases active sites show structural divergences; therefore, this enzyme has been proposed as a potential drug target for selective inhibitors [22,23,24].

Among the dozens of reported drug targets for *T. cruzi* [25], TcTS, TcTR and Tc80 were selected in this work to be studied as molecular targets for STLs.

In this paper, we aim to study the anti-*T. cruzi* properties of two STLs isolated from *Stevia* species. In this sense, in vitro activities against different parasite forms and possible molecular mechanisms of parasite inhibition are explored.

## 2. Materials and Methods

### 2.1. Plant Material

The aerial parts of *Stevia satureiifolia* (Lam.) Sch. Bip. var. *satureiifolia* (Asteraceae) (BAF 744) were collected in Buenos Aires Province, Argentina, in February 2012. The aerial parts of *Stevia alpina* Griseb. (Asteraceae) (BAF 12266) were collected in Catamarca, Argentina, in March 2015. Both species were harvested conservatively to preserve the genetic resource (approx. 10–15% of aerial parts of each plant were cut using scissors). Voucher specimens are available at the Museo de Farmacobotánica, Facultad de Farmacia y Bioquímica, Universidad de Buenos Aires.

### 2.2. Compound Isolation

Dried aerial parts of *S. satureiifolia* var. *satureiifolia* (500 g) were extracted twice by maceration at room temperature with dichloromethane (5 L) for 5 min. After filtration, the extract was dried by vacuum evaporation under reduced pressure, resulting in 18 g of crude extract (3.6% yield). The extract was dewaxed following the protocol described by Elso et al. [26] and fractionated by column chromatography on Silicagel (50 × 4.5 cm, 180 g, 230–400 mesh) using dichloromethane:ethyl acetate (CH_2_Cl_2_:EtOAc) mixtures (100:0–0:100) of increasing polarity. Seven fractions were collected (A–G). The crystalline precipitate that appeared in fraction D [eluted with CH_2_Cl_2_:EtOAc (3:7)] was repeatedly washed with ethyl acetate to produce pure crystals of compound A (0.026% yield based on dry plant material).

The extraction and fractionation of the aerial parts of *Stevia alpina* were carried out as published by de Heluani et al. (1989) [27] to isolate 2-oxo-8-deoxyligustrin (compound B) (0.024% yield based on dry plant material).

The behavior of compounds A and B on thin layer chromatography (TLC) was evaluated using Silicagel 60 F254 as a stationary phase and hexane:EtOAc (1:1) (system I), toluene:EtOAc (1:1) (system II) and CH_2_Cl_2_:EtOAc (4:1) (system III) as mobile phases and anisaldehyde sulfuric acid as a developing reagent. The identities of compounds A and B were determined by proton nuclear magnetic resonance (^1^H-NMR) and carbon nuclear magnetic resonance (^13^C-NMR), heteronuclear single quantum correlation (HSQC), heteronuclear multiple bond correlation (HMBC), correlated spectroscopy (COSY) (Bruker Advance 600) (600 MHz in CDCl_3_), electron impact-mass spectrometry (EI-MS) and infrared spectroscopy (IR) by comparing the spectra obtained with those found in the literature. The purity of both compounds was determined by HPLC using a Varian 9000 instrument equipped with a reversed-phase column Agilent ZORBAX Eclipse Plus C18 (250 mm × 4.6 mm and 5 μm dp), a Rheodyne valve (20 µL) and an UV detector set at 215 nm. Samples were eluted with a gradient of water (A) and acetonitrile (C) from 35% C to 100% C in 30 min. The flow rate was set at 1 mL/min.

### 2.3. Trypanosoma Cruzi In Vitro Assays

#### 2.3.1. Drugs Preparation

Stock solutions of pure compounds A and B (30 mg/mL) were prepared to be diluted as necessary for the biological assays using DMSO as the vehicle. To ensure dissolution, compound solutions in tubes were immersed in an ultrasonic bath (Branson, 2510) at 40 °C for 5 min.

#### 2.3.2. Parasites

Cultures of *Trypanosoma cruzi* epimastigotes (RA strain) were maintained through weekly passages in LIT medium supplemented with 10% fetal bovine serum at 28 °C [28]. *Trypanosoma cruzi* bloodstream trypomastigotes (RA strain) were obtained from infected CF1 mice by cardiac puncture at the peak of parasitaemia 15 days after infection and transfected trypomastigotes expressing β-galactosidase (Clone C4, e Tulahuen strain) were obtained from infected Vero cells [7].

#### 2.3.3. In Vitro Activity Assay against Epimastigotes

The [^3^H]-thymidine uptake assay was performed in order to determine the growth inhibition of *T. cruzi* epimastigotes [29]. The parasite cell density was adjusted to 1.5 × 10^6^ parasites/mL with BHT medium. Compounds A and B were tested at concentrations of 1–100 µg/mL concentrations. Benznidazole was used as a positive control, while DMSO functioned as a negative control for the vehicle. After 72 h incubation, radioactivity was measured as counts per minute (cpm). The percentage of inhibition was calculated as: 100—{[(cpm of treated parasites)/(cpm of untreated parasites)] × 100}

#### 2.3.4. In Vitro Activity Assay against Trypomastigotes

The survival of *T. cruzi* trypomastigotes after treatment with the isolated compounds was evaluated as previously described [7]. Briefly, blood-derived trypomastigotes were incubated with different concentrations of compounds A and B (1–100 µg/mL) for 24 h at 37 °C. DMSO and benznidazole were used as controls for the vehicle and as positive controls, respectively. Live trypomastigotes were counted in a Neubauer chamber. The percentage of live trypomastigotes was calculated as: {[(live parasites after incubation)/(live parasites in untreated wells)] × 100}

#### 2.3.5. In Vitro Activity Assay against Amastigotes

The trypanocidal activity of compounds A and B on intracellular forms of *T. cruzi* was evaluated [6]. Vero cells (5 × 10^3^/well) were infected with culture-derived trypomastigotes of *T. cruzi* Tulahuen strain expressing β-galactosidase (MOI: 10). After an O.N. incubation at 37 °C, and 5% CO_2_, non-infecting parasites were washed out and infected cells were incubated with increasing concentrations of the compounds (0.2–50 µg/mL). Cells treated with DMSO or benznidazole and non-infected cells were included as controls. After 5 days, cells were lysed with 1% Nonidet P-40, and chlorophenol red-β-d-galactopyranoside (CPRG) (100 μM) was added as β-galactosidase substrate. The plate was then incubated for 4 h at 37 °C and β-galactosidase activity was determined by measuring the absorbance at 570 nm. The percentage of inhibition for each compound concentration was calculated as: 100 − {[(absorbance of treated infected cells)/(absorbance of untreated infected cells)] × 100}.

### 2.4. Cytotoxicity Assay

The cytotoxicity was determined by the MTT method [30]. Vero cells were seeded in a flat-bottom 96-well-plate and cultured at 37 °C in a 5% CO_2_ atmosphere in the presence of increasing concentrations of compounds (5–200 µg/mL) or DMSO as a viability control, for 48 h. Then, MTT was added at a final concentration of 0.6 mg/mL and after 2 h incubation at 37 °C, formazan crystals were dissolved with 100 μL of isopropanol. The absorbance was read at 595 nm in a microplate reader (Rayto RT-6000). Results were expressed as the viability percentage of cells: {(absorbance in the presence of the compound)/(absorbance in absence of the compound)} × 100.

### 2.5. TcTR Expression and Purification

*Escherichia coli* BL21 (DE3) cells transformed with the PET28c expression vector and polyHistidine (pHis) constructs encoding for N-terminally hexa-histidine-tagged TcTR were kindly donated by Dr. Carlos Robello. Expression and purification were performed as described by Arias et al. [31]. Isopropyl β-D-1-thiogalactopyranoside (IPTG) was added at a final concentration of 0.5 mM to overnight cultures of transformed *E. coli* grown to exponential phase (OD_600_ of 0.6). After 20 h incubation with orbital shaking at 20 °C, cells were harvested by centrifugation (5000× *g*, 10 min, 4 °C). The bacterial pellet was resuspended in buffer A (20 mM Tris–HCl, pH 7.5, 400 mM NaCl, 10 mM imidazole) and 1 mM FAD cofactor. The cell suspension was lysed using an Ultrasonic homogenizer (JY 92-IN) and debris was removed by centrifugation and filtration. The obtained sample was loaded onto a 1 mL HisTrap^TM^ HP (GE Healthcare). The column was washed with buffer A and 30 mM imidazole, and the recombinant protein was eluted with 100% buffer B (20 mM Tris–HCl, pH 7.5, 400 mM NaCl, 300 mM imidazole) in 10 column volumes. After analysis by SDS-PAGE, the fractions containing the pure enzyme were pooled. Protein concentration was determined from the absorbance of the flavin prosthetic group at 458 nm (ε_458_= 11.2 mM^−1^ cm^−1^) [32], with a Cary 60 UV-Vis spectrophotometer (Agilent Technologies).

### 2.6. Enzymatic Assays

#### 2.6.1. TcTR Activity Assay

TcTR activity was measured by monitoring DTNB reduction at 405 nm in a coupled assay that guaranteed the oxidation of T(SH)_2_ [31]. The reaction mixture contained 20 mM Tris–HCl, pH 7.5, 1 mM EDTA, 200 μM NADPH, 4 nM TcTR, 12 μM T(SH)_2_, 200 μM DTNB, and 50 μM of compound A or compound B. Reactions were carried out at 25 °C in a final volume of 100 μL in a 96-well plate. Absorbance was measured in a Varioskan Flash (Thermo scientificTM, Waltham, MA, USA) plate reader with kinetic mode. Initial reaction velocity (Vi) was determined by fitting the initial part of each run by linear regression.

#### 2.6.2. TcTS Activity Assay

Recombinant TcTS was expressed and purified as described by Buschiazzo et al., 1996 [33]. The fluorescence emitted by the 4-methylumbelliferone obtained in the hydrolysis of 4-Methylumbelliferyl-N-acetyl-α-D-Neuraminic Acid (sodium salt) (MUNANA) (Biosynth Carbosynth) was measured in order to determine the sialidase activity of the purified recombinant TcTS. The reaction mixture, with a final volume of 50 µL contained 150 mM NaCl and 50 mM Tris-ClH pH 6.8. TcTS was initially incubated 15 min with 100 µM of either compound A or B. DMSO was used as a negative control. MUNANA was added at 0.2 mM concentration. Fluorescence was measured for 30 min at 25 °C in a microplate reader (FilterMax F5 Multi-Mode Microplate Reader).

#### 2.6.3. Tc80 Activity Assay

The expression and purification of Tc80 were performed as described by Bivona et al., 2018 [34]. Recombinant Tc80 (0.02 µg) was incubated for 5 min with increasing concentrations of compounds A or B (12.5–100 µM). DMSO was used as a negative control. Prolyl oligopeptidase activity was evaluated by monitoring the conversion of the fluorogenic substrate dipeptide Z-Gly-Pro-7-amido-4-methyl coumarin (Bachem) to the 7-amino-4-methyl coumarin (AMC) product (λ_excitation_ = 355 nm, λ_emission_ = 460 nm). Fluorescence was measured at 37 °C in a final volume of 100 μL reaction buffer (25 mM Tris, 250 mM NaCl, 2.5 mM DTT, pH 7.5) in a 96-well black plate (Costar Corning) on a PerkinElmer Victor^3^ fluorometer. From the AMC formation over time, relative fluorescence units (RFU) recorded were used to calculate the initial reaction velocity (Vi) as the slope (ΔRFU/Δtime) of the linear region in the RFU vs. time curve for each compound.

To construct the Michaelis-Menten plot, Tc80 Vi was determined for different substrate concentrations (2–64 µM) in the presence or absence of 50 µM of compound A.

### 2.7. Molecular Docking Studies

#### 2.7.1. Molecular Modeling of Tc80 as a Proposed Target

The 3D structure of the prolyl endopeptidase Tc80 from *T. cruzi* has not yet been elucidated. In this sense, a homology modelling of the primary sequence Q71MD6 entry of Uniprot https://www.uniprot.org/ (accessed on 1 November 2022)—the prolyl endopeptidase of 80 kDa from *T. cruzi*, with organism identifier OX = 5693 and gene name GN = TCPO — was performed in the Swiss Model [35] server using the 1H2W PDB entry (https://www.rcsb.org/ accessed on 1 November 2022) as the selected template. The selected template is a prolyl oligopeptidase from the porcine brain with 43.6% sequence identity with the Tc80 protein. To achieve a reliable model, the global model quality and QMEANDistCo [36] parameters were considered, and the rotamer outliers observed in the Ramachandran plot were corrected.

#### 2.7.2. Molecular Modeling of Proposed Ligands for Tc80

The structures of compounds A and B were built and checked with MarvinSketch version 21.17.0 (Chemaxon, Budapest, Hungary) and GaussView [37] softwares (Gaussian, Inc., Wallingford, CT, USA). The ligands ground state employed in the docking experiments were obtained by performing a geometry optimization with a semi-empirical AM1 method and then, were refined within the density functional theory with use of the B3LYP [38] functional and 6-31G* basis set. The partial atomic charges were obtained through a single point at HF/6-31G* using Gaussian 03 and the Merz–Singh–Kollman protocol [39,40].

#### 2.7.3. Docking Studies

A blind docking was carried out by mapping the entire surface of the Tc80 protein with the Autodock Vina software (The Scripps Research Institute, La Jolla, CA, USA) [41]. The grid box with the corresponding coordinates of the box’s spatial origin was checked in the AutoDockTools 1.5.6 software (The Scripps Research Institute, La Jolla, CA, USA) for both ligands. The protein surface was mapped with two boxes of 52 Å^3^ in the search for the docking site. The site of higher docking binding energy was re-gridded with a smaller box centered in each ligand and each of them was re-docked with a higher exhaustiveness (=1000) to thoroughly evaluate the best docking pose for both ligands. Default values for the other configuration parameters were used. Interaction energy results were expressed as ΔG_dock_, in Ikcal/mol. Interactions of the ligands with the target were evaluated with the Pymol 2.4.0 software (Schrödinger, Inc., New York, NY, USA).

### 2.8. Transmission Electron Microscopy

All procedures were carried out according to Brengio et al. [42]. Briefly, epimastigotes from *T. cruzi* were treated with different concentrations of compound A for 48 h or 72 h. Then, the parasites were centrifuged at 1000× *g* for 10 min and fixed with 2.5% glutaraldehyde. Subsequently, they were washed three times with PBS and postfixed overnight with 2% OsO_4_. After washing twice with PBS, cells were stained with 1% uranyl acetate. The samples were dehydrated sequentially in ethanol and acetone and embedded in Epon 812. Ultrathin slices of 60 nm were obtained using an automatic Leica-ultracut R ultramicrotome and observed with a Microscope Zeiss EM900.

### 2.9. Statistical Analysis

The results are expressed as means ± SEM. The GraphPad Prism 8.0 software (GraphPad Software Inc., San Diego, CA, USA) was employed to determine IC_50_ and CC_50_ values by non-linear regression.

Student’s *t* test, or one-way ANOVA and Dunnett’s post-test were used for the determination of statistical significance. *p*-values < 0.05 were considered significant.

## 3. Results

### 3.1. Compounds Isolation

**Figure 1:**
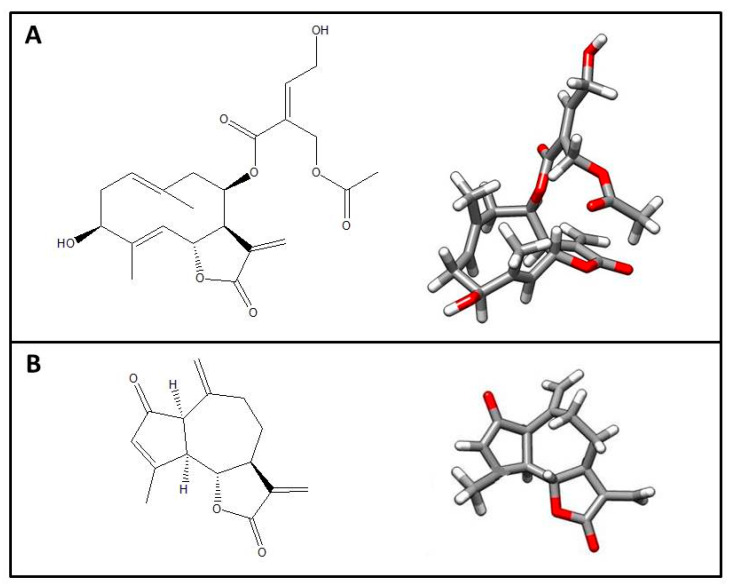
Two- and three-dimensional structures of isolated compounds: (**A**) santhemoidin C; (**B**) 2-oxo-8-deoxyligustrin.

The sesquiterpene lactones (STLs), santhemoidin C (Compound A) and 2-oxo-8-deoxyligustrin (Compound B), were isolated from the dichloromethane extracts of *S. satureiifolia* var. *satureiifolia* and *S. alpina*, respectively (Figure 1). The STLs were identified by spectroscopic methods and compared with spectra found in literature [26,27,43]. The TLC assessment of the STLs afforded Rf values of 0.14 and 0.57 (system I), 0.21 and 0.67 (system II) and 0.10 and 0.78 (system III), for santhemoidin C and 2-oxo-8-deoxyligustrin, respectively. The purity of the isolated compounds, as determined by HPLC, was 99.3 and 95.3%, respectively.

### 3.2. Anti-Trypanosoma Cruzi Activity

**Figure 2:**
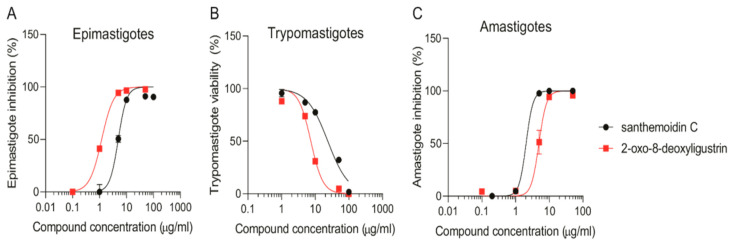
Effect of isolated compounds on the three stages of *T. cruzi*. Both santhemoidin C and 2-oxo-8-deoxyligustrin were tested at 1–100 µg/mL concentrations in duplicates to evaluate: (**A**): Growth inhibition of *T. cruzi* epimastigotes. (**B**) Trypanocidal activity against bloodstream trypomastigotes; (**C**) inhibition of *T. cruzi* amastigotes. Results are expressed as the mean ± SEM.

The effect of the isolated STLs was evaluated in vitro against epimastigotes, trypomastigotes and amastigotes of *T. cruzi* (Figure 2) (Table 1).

Santhemoidin C and 2-oxo-8-deoxyligustrin were active against epimastigotes, reaching IC_50_ values of 4.96 ± 0.47 and 1.20 ± 0.06 µg/mL (11.80 and 4.98 µM), respectively (Figure 2A). When tested against bloodstream trypomastigotes, both compounds decreased the number of live parasites in a concentration-dependent manner. Both compounds showed moderate activity with IC_50_ values of 23.58 ± 3.03 and 6.39 ± 0.70 µg/mL (56.08 and 26.19 µM), respectively (Figure 2B). The STLs were also tested on the intracellular form of *T. cruzi*, resulting in significant inhibition of amastigote replication with IC_50_ of 2.05 ± 0.002 and 4.93 ± 0.52 µg/mL (4.88 and 20.20 µM) (Figure 2C). Results of the effect of the reference drug benznidazole on the three forms of *T. cruzi* are shown in Figure S1 (Supplementary materials).

### 3.3. Cytotoxicity

**Figure 3:**
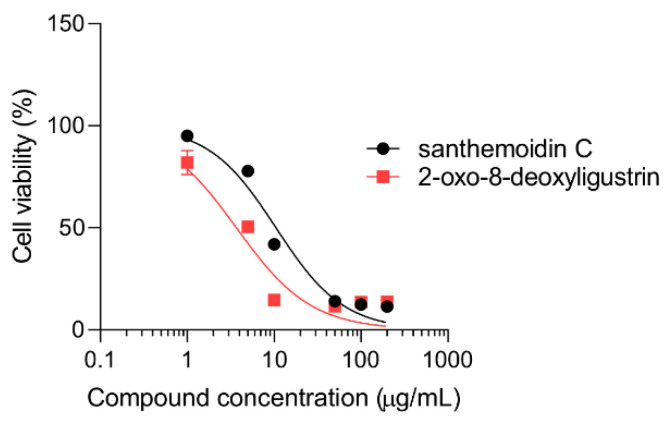
Cytotoxicity evaluation of the STLs on mammalian cells. Vero cell viability was determined by the MTT method in the presence of either santhemoidin C or 2-oxo-8-deoxyligustrin. Cells were incubated for 48 h with increasing concentrations of the compounds. Results are expressed as a percentage of viability. Bars represent the means ± SEM.

The cytotoxic effect of santhemoidin C and 2-oxo-8-deoxyligustrin was assessed in vitro on Vero cells. The STLs respectively presented CC_50_ values of 11.03 ± 0.30 µg/mL (26.23 µM) and 3.81 ± 0.84 µg/mL (15.61 µM) (Figure 3). Santhemoidin C showed selectivity against the intracellular form of *T. cruzi*. The selectivity index (calculated as the relation between CC_50_ and IC_50_) was 5.38.

### 3.4. Effect of the Compounds on TcTR, TcTS and Tc80

The inhibition of TcTR and TcTS enzymatic activities was assessed in vitro, as previously indicated, for each compound concentration in triplicate. Results indicated that neither santhemoidin C nor 2-oxo-deoxyligustrin inhibited TcTR or TcTS.

**Figure 4:**
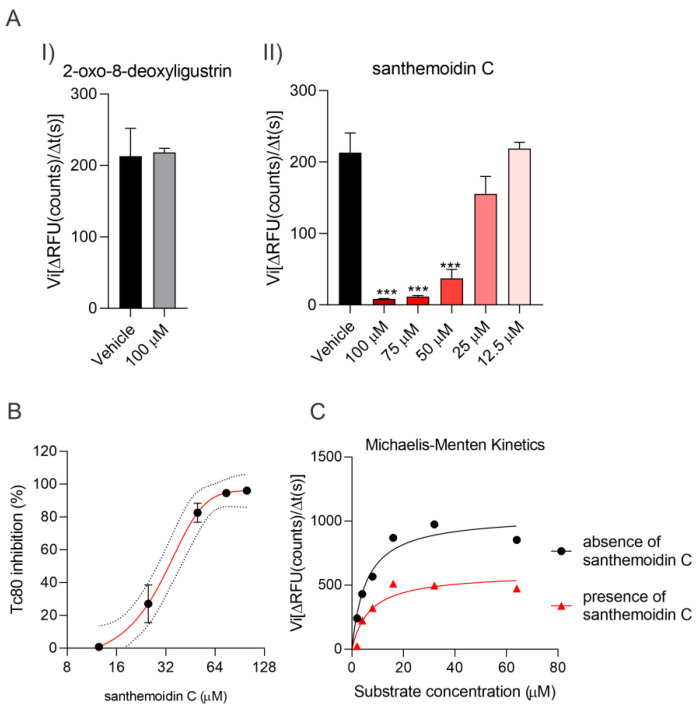
Enzymatic activity of Tc80 was measured by the conversion of the fluorogenic substrate Z-Gly-Pro-7-AMC to AMC in the presence of santhemoidin C or 2-oxo-8-deoxyligustrin. (**A**) Initial reaction velocity (Vi) was determined as the slope (ΔRFU/Δtime) from the linear region in the RFU vs. time curve for tested concentrations of santhemoidin C (**I**) or 2-oxo-8-deoxyligustrin (**II**). (**B**) Dose-dependent inhibition of Tc80 (0.02 µg) by santhemoidin C. (**C**) Michaelis-Menten Plot was constructed by non-linear regression to estimate kinetic parameters (Km and V_max_) in the presence or absence of 50 µM santhemoidin C. Results are expressed as the mean ± SEM. Asterisk indicates significant differences compared to vehicle (*** *p* < 0.001).

In relation to Tc80, the STL 2-oxo-8-deoxyligustrin was not able to inhibit this enzyme Figure 4(AI). On the other hand, incubation with increasing concentrations of santhemoidin C resulted in enzymatic inhibition of Tc80 Figure 4(AII), reaching an IC_50_ value of 34.90 ± 5.90 µM (Figure 4B).

In order to explore possible molecular mechanisms of inhibition, a Michaelis–Menten plot was constructed in the presence or absence of santhemoidin C. Non-linear regression was applied to calculate the kinetic constants K_M_ (Michaelis constant) and V_max_ (maximum velocity), resulting in values of 6.6 µM and 602 RFU/s, respectively, in the presence of santhemoidin C at 50 µM, while the reaction without santhemoidin C provided a V_max_ of 1056 RFU/s and a K_M_ of 5.9 µM (Figure 4C). The reduction of V_max_ together with an unchanged value of the K_M_ constant in the presence of santhemoidin C suggests a non-competitive inhibition.

### 3.5. Docking Studies

A molecular docking study of santhemoidin C and 2-oxo-8-deoxyligustrin on the prolyl oligopeptidase of 80 kDa of *T. cruzi* (Tc80) was performed.

**Figure 5:**
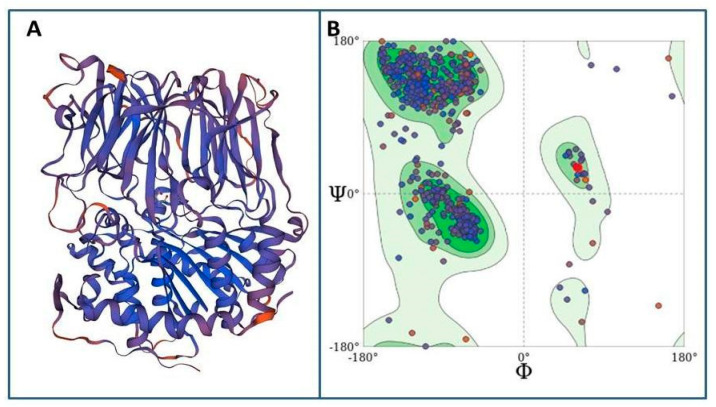
Homology modeling of Tc80. (**A**) Graphic representation of the 3D structure of the Tc80 protein of *T. cruzi.* The color pattern is in accordance with the quality of the estimated local model: in blue the similarity with the template is higher, and in red, the similarity is lower. (**B**) Ramachandran plot showing the ϕ and Ψ dihedral values for each amino acid of the Tc80 model.

Primary sequence homology modeling of Tc80 was performed with the Swiss Model server using a porcine brain prolyl oligopeptidase, with 43.6% sequence identity to the Tc80 target, as a template (Figure 5A). The model had a Global Model Quality Estimate of 0.81 and a QMEANDistCo of 0.79 ± 0.05. 93.65% of the residues were distributed in Ramachandran’s favored regions, and the rotamer outliers were corrected (Figure 5B).

**Figure 6:**
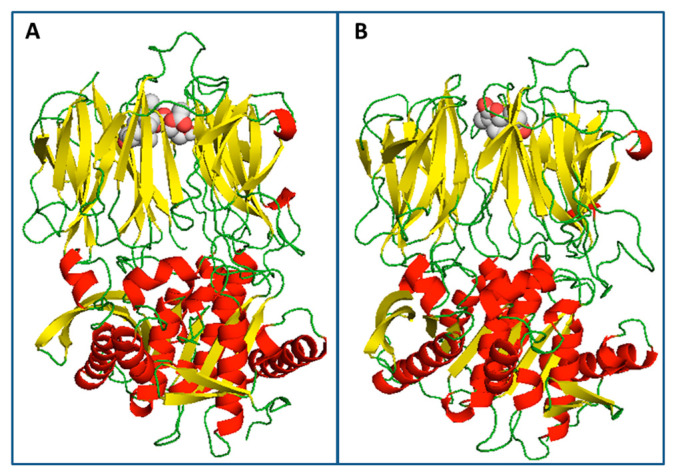
(**A**) Best interaction pose for santhemoidin C (light gray and red spheres) with Tc80 (**B**) Best interaction pose for 2-oxo-8-deoxyligustrin (light grey and red spheres) with Tc80. The secondary structure of the protein is rendered in cartoon representation, as: α-helices (red), 𝛽-sheets (yellow) and loops and turns (green).

A blind molecular docking performed on the entire surface of the Tc80 model of *T. cruzi* identified a preferential docking site for both ligands, as was shown in Figure 6.

**Figure 7:**
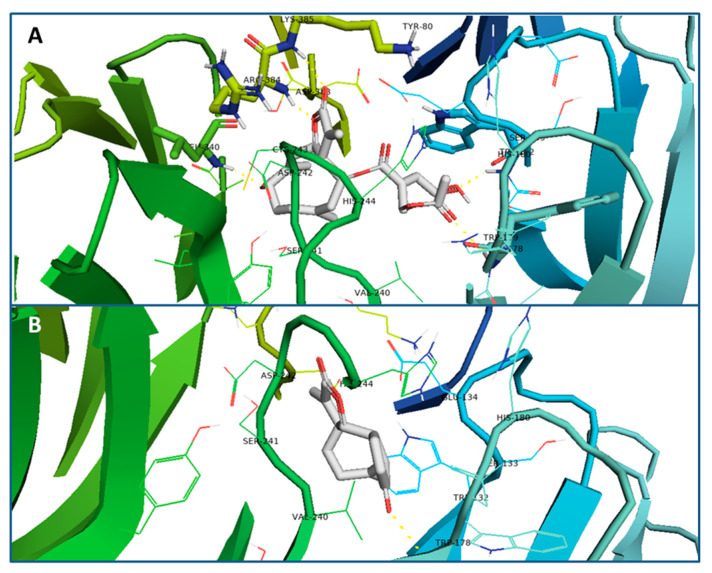
(**A**) Best interaction pose for santhemoidin C (in gray) with target Tc80. Five hydrogen bonds can be observed (yellow lines) with residues Trp 178, Trp 132, Lys 385, Leu 340 and Arg 384. (**B**) Best interaction pose for 2-oxo-8-deoxyligustrin (in grey) with target Tc80. One hydrogen bond formed can be observed with residue Trp178 (yellow lines).

Results showed ΔG_dock_ energy values for santhemoidin C and 2-oxo-8-deoxyligustrin of −8.5 and −7.1 kcal/mol, respectively. The best poses of interaction for each compound resulted in the formation of five hydrogen bonds for the first compound (Figure 7A) and only one for the last one (Figure 7B).

### 3.6. Effect on Parasite Ultrastructure

**Figure 8:**
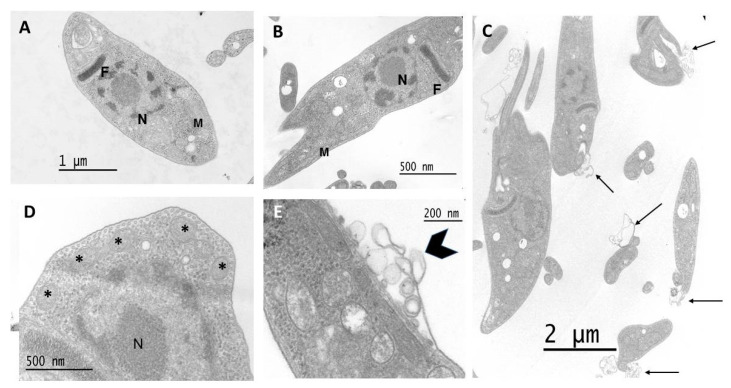
(**A**,**B**) Untreated parasites displayed typical morphology. M, mitochondrion; K, kinetoplast; N, nucleus. (**C**) Epimastigotes treated for 48 h with 12 µM of santhemoidin C induced a plasma membrane shedding (black arrows). (**D**) Parasites treated with 18 µM for 48 h displayed the formation of cytoplasmic vacuoles, which resemble autophagosomes (asterisks) and (**E**) a detachment of plasma membrane (arrowhead).

The effect of santhemoidin C on the epimastigote form of *T. cruzi* at the ultrastructural level was analyzed using transmission electron microscopy. Untreated parasites displayed typical morphology: a single nucleus (N), a kinetoplast (K) near the flagellum (F), and a single branched mitochondrion (M) (Figure 8A,B). The treatment with santhemoidin C with 12 µM for 48 h promoted plasma membrane shedding (Figure 8C, black arrows). Higher doses (18 µM) caused the formation of cytoplasmic vacuoles, which resemble autophagosomes (Figure 8D, asterisks), as well as the detachment of the plasma membrane (Figure 8E, arrowhead). Similar phenotypes were observed at 72 h of treatment.

## 4. Discussion

We have previously reported the trypanocidal activity of an organic extract of *S. satureiifolia* var. *satureiifolia* and the isolation of bioactive phenolic compounds from this species [28]. In the search for novel trypanocidal hit compounds, in this work a dichloromethane extract of *S. satureiifolia* var. *satureiifolia* was fractionated and purified by chromatographic techniques, leading to the isolation of a sesquiterpene lactone, which was identified as santhemoidin C. This sesquiterpene lactone has been reported to be present in *Urolepis hecatantha* (DC) R. M. King and H. Rob and *Skuria* spp. (Asteraceae) [26,44], but this is the first time that this compound has been reported in the genus *Stevia*.

In this paper, we continued studying the phytochemistry of the species *S. alpina*, aiming to find new antiparasitic compounds. The fractionation and purification of the organic extract led to the isolation of the STL 2-oxo-8-deoxyligustrin, which has been previously reported in *S. alpina.*

Both STLs, santhemoidin C and 2-oxo-8-deoxyligustrin, were evaluated against *T. cruzi*, the causative agent of Chagas disease or American trypanosomiasis. Initially, the compounds were tested on epimastigotes, which is the non-infective and replicative form of *T. cruzi.* The STLs exerted an effect on this form of the parasite with IC_50_ values of 11.80 and 4.98 µM, respectively. In the search for new trypanocidal compounds, both STLs were evaluated against the infective and intracellular forms of the parasite. The STLs were moderately active against trypomastigotes. However, santhemoidin C significantly inhibited the replication of amastigotes with an IC_50_ value of 4.88 µM. This is an interesting result since amastigotes represent the replicative form of the parasite and therefore their elimination is relevant in the course of the infection in humans. The cytotoxicity of the compounds was evaluated in mammalian cells. Santhemoidin C showed better selectivity to intracellular parasites compared to 2-oxo-8-deoxyligustrin. Given the data presented in this paper, santhemoidin C and 2-oxo-8-deoxyligustrin are suitable candidates for the obtention of semisynthetic derivatives in the search for more effective and less cytotoxic trypanocidal compounds. In this sense, the synthesis of ester derivatives of STLs bearing an OH group, such as santhemoidin C, has been an alternative to reduce cytotoxicity and improve the selectivity index [6].

In an attempt to elucidate the potential molecular targets of the STLs, the effect of the two isolated compounds on three relevant enzymes of *T. cruzi*: TcTR, TcTS and Tc80, was evaluated. Santhemoidin C and 2-oxo-8-deoxyligustrin did not affect TcTR and TcTS activities. Regarding Tc80, santhemoidin C proved to be an inhibitor of enzymatic activity in vitro (IC_50_ = 34.90 µM). On the other hand, 2-oxo-8-deoxyligustrin showed no activity against Tc80 under the conditions evaluated. The results obtained for santhemoidin C could be related to those found with a terpenoid-rich extract of *Clethra fimbriata* [45]. According to the authors, the trypanocidal effect of the extract and the decrease in trypomastigote infectivity may be related to the effect on proteins involved in the adhesion and invasion processes, such as Tc80 oligopeptidase, among others. From the Michaelis–Menten plot constructed, the kinetic constants were calculated, resulting in a decrease in the V_max_ while the K_M_ value was not affected. According to enzyme kinetics theory, a reduction in V_max_ is associated with a non-competitive inhibition mechanism.

As it was specified earlier, the tridimensional structure of Tc80 has not been elucidated, although extensive research has been published on it from in silico studies [23,46]. It has been described that this enzyme is composed of a polypeptide chain with two identifiable domains. The catalytic domain exhibits a characteristic α/β-hydrolase fold and contains a central β-sheet, constituted of eight strands, surrounded by five helices. The second domain corresponds to the non-catalytic domain which is composed of a set of seven similar β-sheets that collectively form a β-propeller structure that is twisted and radially positioned around a central axis forming a central cavity as a funnel. Bastos et al. [23], performed molecular dynamics and docking calculations in which it was determined that the collagen substrate would access the Tc80 active site through the interface region located between the two domains. Moreover, these authors suggested that the β-propeller structures present in the non-catalytic domain are involved in multiple protein–protein interactions and consequently are essential for the cleavage of the collagen substrate.

In the present work, the binding characteristics of santhemoidin C to the Tc80 protein were investigated through molecular docking studies to deepen the understanding of its molecular mechanism of inhibition. In the predicted docked conformation, its hydroxyl, carbonyl and ester groups were shown to be hydrogen bond acceptors for five amino acid residues of the non-catalytic domain of Tc80. In contrast, 2-oxo-8-deoxyligustrin only formed one hydrogen bond. Furthermore, the docking energy calculated for santhemoidin C was higher (ΔG_dock_: −8.5 kcal/mol) than the one registered for 2-oxo-8-deoxyligustrin (ΔG_dock_: −7.1 kcal/mol). This data is consistent with the in vitro results in which 2-oxo-8-deoxyligustrin was not shown to inhibit enzymatic activity, which can be explained by the weak interaction observed with the Tc80 target in the docking calculations, whereas santhemoidin C strongly inhibited enzyme activity in vitro due to a more stable interaction observed in silico. In addition, the proposed inhibition mechanism that was inferred from the Michaelis-Menten plot as a non-competitive inhibitor is supported by the fact that santhemoidin C interacts with the non-catalytic domain and does not appear to be directly bonding to the active site or the substrate interaction site of the enzyme. Considering these results, we hypothesized that modulation of the structure of this domain by santhemoidin C somehow affects the ability of interaction with the substrate, explaining the inhibition as well as providing a novel mechanism of action for the study and design of potential trypanocidal molecules.

Finally, to further explore the effect of santhemoidin C on *T. cruzi*, an ultrastructural analysis was performed by transmission electron microscopy. Cytoskeleton-affecting drugs can induce the formation of membrane protrusions resembling surface blebs, which may progress to membrane shedding [47]. The ultrastructural studies displayed plasma membrane blebbing and autophagosome-like structures in STLs-treated parasites, and this could be indicative that different cell death mechanisms are being triggered by the STLs. Comparably to santhemoidin C, the STL cynaropicrin showed plasma membrane shedding on *T. cruzi*, as demonstrated by transmission electron microscopy analysis [48]. Other STLs such as 8-epi-xanthatin-1β,5β-epoxide and inuloxin A affect the plasma membrane permeability [49]. Similarly, lactones isolated from *Nectandra barbellata* have shown the same effect [50]. The nonpolar characteristic of STLs could determine the effects on the plasma membrane, producing alterations and consequently leading to intracytoplasmic effects in the parasite. However, the mechanism behind the membrane alteration induced by STLs needs to be further studied.

These results together suggest that santhemoidin C probably has multiple independent ways of exerting its anti-*T. cruzi* effects. At least two of them were demonstrated in the present manuscript: on the one hand, the inhibition of Tc80, and on the other, the plasma membrane alteration.

## 5. Conclusions

In this work, the anti-*T. cruzi* effect of the two natural STLs, sesquiterpene lactones, santhemoidin C and 2-oxo-8-deoxyligustrin isolated from *Stevia* spp., was evaluated. Santhemoidin C was shown to be more active and selective than 2-oxo-8-desoxyligustrin on amastigotes. The first showed inhibition of Tc80 and promoted plasma membrane shedding by the parasites. These results show the relevance and importance of natural products in the discovery and identification of novel trypanocidal hit compounds for the development of possible therapies for Chagas disease.

## Figures and Tables

**Table 1:** IC_50_ values calculated for each compound against the three forms of *T. cruzi*.

Compounds	IC_50_ Values [µg/mL +/− SD (µM)]		
	Epimastigotes	Trypomastigotes	Amastigotes
santhemoidin C	4.96 ± 0.47 (11.80)	23.58 ± 3.03 (56.08)	2.05 ± 0.002 (4.88)
2-oxo-8-deoxy-ligustrin	1.20 ± 0.06 (4.98)	6.39 ± 0.70 (26.19)	4.93 ± 0.52 (20.20)

## Data Availability

Not applicable.

## Supplementary Materials

The following supporting information can be downloaded at: https://www.mdpi.com/article/10.3390/pharmaceutics15020647/s1, Figure S1: Trypanocidal activity of the reference drug benznidazole determined by in vitro assays against epimastigotes, trypomastigotes and amastigotes of *T*. *cruzi*.
