# Supplementary material for: Anti-Trypanosoma cruzi Properties of Sesquiterpene Lactones Isolated from Stevia spp.: In Vitro and In Silico Studies
Source: Pharmaceutics. 2023 Feb 15;15(2):647. doi: 10.3390/pharmaceutics15020647 (PMC9961625; doi:10.3390/pharmaceutics15020647)
Supplement: Supplementary file 1 [file pharmaceutics-15-00647-s001.zip › pharmaceutics-2142360-supplementary.pdf]

pharmaceutics

Article

Supplementary materials: Anti-*Trypanosoma cruzi* Properties of Sesquiterpene Lactones Isolated from *Stevia* spp.: In Vitro and In Silico Studies

Jimena Borgo [1,2], Orlando G. Elso [2,3], Jessica Gomez [4], Mauro Coll [4], Cesar A. N. Catalán [5], Juan Mucci [6], Guzmán Alvarez [7], Lía M. Randall [7], Patricia Barrera [4], Emilio L. Malchiodi [8,9], Augusto E. Bivona [8,9,†], María Florencia Martini [1,10,†] and Valeria P. Sülsen [1,2,*,†]

1 Instituto de Química y Metabolismo del Fármaco (IQUIMEFA), CONICET-Universidad de Buenos Aires, Junín 956, piso 2, Buenos Aires C1113AAD, Argentina
2 Cátedra de Farmacognosia, Facultad de Farmacia y Bioquímica, Universidad de Buenos Aires, Junín 956, piso 2, Buenos Aires C1113AAD, Argentina
3 Unidad de Microanálisis y Métodos Físicos Aplicados a Química Orgánica (UMYMFOR), Facultad de Ciencias Exactas y Naturales, CONICET-Universidad de Buenos Aires, Ciudad Universitaria, Pabellón 2, piso 3, Buenos Aires C1428EGA, Argentina;
4 Facultad de Ciencias Médicas, Instituto de Histología y Embriología "Dr. Mario H. Burgos" (IHEM), Universidad Nacional de Cuyo-CONICET, CC 56, Mendoza 5500, Argentina
5 Instituto de Química Orgánica, Facultad de Bioquímica, Química y Farmacia, Universidad Nacional de Tucumán, Ayacucho 471, San Miguel de Tucumán, Tucumán T4000INI, Argentina
6 Instituto de Investigaciones Biotecnológicas, Universidad Nacional de San Martín-CONICET, Buenos Aires 5500, Argentina
7 Laboratorio de Moléculas Bioactivas, Departamento de Ciencias Biológicas, CENUR Litoral Norte, Universidad de la República, Paysandú 60000, Uruguay
8 Cátedra de Inmunología, Facultad de Farmacia y Bioquímica, Universidad de Buenos Aires, Junín 956, piso 4, Buenos Aires C1113AAD, Argentina
9 Instituto de Estudios de la Inmunidad Humoral (IDEHU), CONICET-Universidad de Buenos Aires, Junín 956, piso 4, Buenos Aires C1113AAD, Argentina
10 Cátedra de Química Medicinal, Facultad de Farmacia y Bioquímica, Universidad de Buenos Aires, Junín 956, Planta Principal, Buenos Aires C1113AAD, Argentina
* Correspondence: vsulsen@ffyb.uba.ar; Tel.: +54-11-5287-4272
† The work was co-directed by the three authors.

Citation: Borgo, J.; Elso, O.G.; Gomez, J.; Coll, M.; Catalán, C.A.N.; Mucci, J.; Alvarez, G.; Randall, L.M.; Barrera, P.; Malchiodi, E.L.; et al. Anti-*Trypanosoma cruzi* Properties of Sesquiterpene Lactones Isolated from *Stevia* spp.: In Vitro and In Silico Studies. *Pharmaceutics* **2023**, *15*, 647.https://doi.org/10.3390/pharmaceutics15020647

Academic Editor: Maria João Castro Gouveia

Received: 20 December 2022
Revised: 3 February 2023
Accepted: 7 February 2023
Published: 15 February 2023

Copyright: © 2023 by the authors. Licensee MDPI, Basel, Switzerland. This article is an open access article distributed under the terms and conditions of the Creative Commons Attribution (CC BY) license (https://creativecommons.org/licenses/by/4.0/).

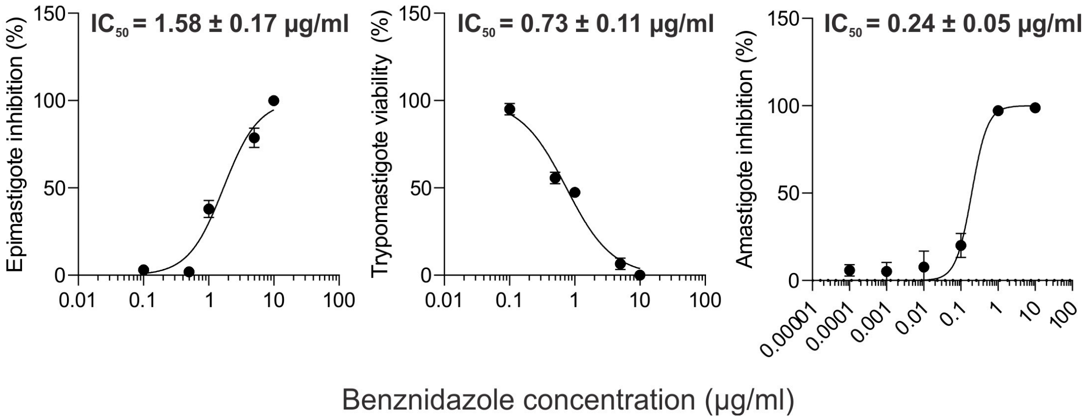

Figure S1. Trypanocidal activity of the reference drug benznidazole determined by *in vitro* assays against epimastigotes, trypomastigotes and amastigotes of *T. cruzi*.
